# Bioavailability of the calcium salt of dl-methionine hydroxy analog compared with dl-methionine for nitrogen retention and the preference of nursery pigs for diets based on the 2 forms of methionine

**DOI:** 10.1093/jas/skaa349

**Published:** 2020-10-28

**Authors:** Minqi Q Wang, La T T Huyen, Jung W Lee, Sheila H Ramos, John K Htoo, La V Kinh, Merlin D Lindemann

**Affiliations:** 1 Department of Animal and Food Sciences, University of Kentucky, Lexington; 2 Institute of Agricultural Sciences for Southern Vietnam (IASVN), Ho Chi Minh City, Vietnam; 3 Evonik (SEA) Pte. Ltd., #07-18 Nordic European Centre, Singapore; 4 Evonik Operations GmbH, Hanau-Wolfgang, Germany

**Keywords:** bioavailability, diet preference, methionine source, pigs

## Abstract

Experiments were conducted to determine the relative bioavailability (**RBV**) of the calcium salt of the hydroxy analog of dl-methionine (**MHA-Ca**, 84%) to dl-methionine (dl**-Met**, 99%) as Met sources fed to pigs. In experiment 1, 42 crossbred barrows (initial BW of 15.0 ± 0.7 kg) were allotted to 7 treatments in an N-balance study. The basal diet (**BD**) was formulated to contain 15.4% CP and 0.22% Met (70% of requirement). Diets included (1) BD, (2) BD + 0.025% dl-Met, (3) BD + 0.050% dl-Met, (4) BD + 0.075% dl-Met, (5) BD + 0.038% MHA-Ca, (6) BD + 0.077% MHA-Ca, and (7) BD + 0.115% MHA-Ca. An increase in dietary inclusion rates of both Met sources linearly increased (*P <* 0.01) N retained (g/d) and N retention (% of intake). Using linear slope-ratio regression, the RBV value of MHA-Ca to dl-Met for N retained (g/d) was 63.0% on a product-to-product basis (75.0% on an equimolar basis). In experiment 2, 40 crossbred barrows (initial BW of 15.5 ± 1.5 kg) were allotted to 5 treatments in another N-balance study. The BD was formulated to contain 17.0% CP and 0.22% Met (70% of requirement). Diets included (1) BD, (2) BD + 0.030% dl-Met, (3) BD + 0.060% dl-Met, (4) BD + 0.046% MHA-Ca, and (5) BD + 0.092% MHA-Ca. Increasing levels of dl-Met or MHA-Ca increased N retained (g/d) and N retention (% of intake) linearly (*P* < 0.001) and quadratically (*P* < 0.05). Using linear slope-ratio regression, a product-to-product RBV value of MHA-Ca to dl-Met was 68.4% (81.4% on an equimolar basis) for N retained (g/d). In experiment 3, 276 pigs (12 barrow and 11 gilt replicates; initial BW of 7.09 ± 1.1 kg) were used in 3 diet preference studies. Pigs were randomly allotted to 1 of 3 treatment comparisons of feed choice: (1) BD (0.23% Met) or BD + 0.07% dl-Met; (2) BD or BD + 0.0825% MHA-Ca, and (3) BD + 0.07% dl-Met or BD + 0.0825% MHA-Ca. Pigs consumed a higher percentage (55 vs. 45%; *P* = 0.008) of their total feed intake from the diet supplemented with 0.07% dl-Met in Comparison 1, but a lower percentage (45 vs. 55%; *P* = 0.003) of their total feed intake from the diet supplemented with 0.0825% MHA-Ca in Comparison 2. There was no diet preference for dl-Met or MHA-Ca in Comparison 3. The observed Met source preference differences occurred in the barrow replicates but not in the gilt replicates. These results demonstrated the mean RBV of MHA-Ca to dl-Met of 65.7% on a product-to-product (wt/wt) basis or 78.2% on an equimolar basis and that a preference for Met sources was observed in barrows but not in gilts.

## Introduction

Methionine is a limiting sulfur-containing amino acid (AA) in conventional corn–soybean meal-based diets fed to pigs (Cromwell, 2004). Crystalline dl-methionine (dl**-Met**, 99%), a highly available Met source, is widely supplemented to Met-deficient diets to optimize growth of pigs. Alternatively, the calcium salt of the hydroxy analog of dl-Met (**MHA-Ca**, 84%) and liquid dl-Met hydroxy analog-free acid (**MHA-FA,** 88%) are utilized as alternative Met sources in swine diets to improve N retention by pigs.

Most of the published studies dealing with the relative bioavailability (**RBV**) of Met sources in pigs have been focused on liquid MHA-FA. For instance, Kim et al. (2006) reported the RBV of MHA-FA compared with dl-Met of 65.2% on a product-to-product basis (74.1% on an equimolar basis) for N retained per day (g) in growing pigs weighing from 16 to 21 kg. Also, Shoveller et al. (2010) demonstrated that the biological efficacy for MHA-FA compared with dl-Met in growing pigs was 65.7% on a product-to-product basis (74.4% on an equimolar basis) for protein deposition using the indicator AA oxidation method in a slope-ratio comparison.

While Opapeju et al. (2012) reported the bioavailability of MHA-Ca relative to dl-Met of 71.2% on a product-to-product basis (84.8% on an equimolar basis) for N retention rate expressed as % of intake in growing pigs weighing from 18 to 22 kg, more information about the RBV of MHA-Ca to dl-Met in pigs is needed to provide a more robust estimate of the RBV of MHA-Ca compared with dl-Met in pigs. We hypothesized that the RBV of MHA-Ca would be lower than that of dl-Met for N retention of starter pigs.

Regarding dietary preference of nursery pigs for Met levels and sources, Roth et al. (2006) demonstrated that nursery pigs exhibit a preference for Met-fortified diets over Met-deficient diets. Ettle et al. (2010) reported that nursery pigs prefer to consume more of the Met-deficient diets supplemented with dl-Met than the MHA-FA-supplemented diets regardless of the inclusion level of MHA-FA. We, thus, further hypothesized that if a preference for Met-sources existed, that the preference observed would similarly be for greater consumption of dl-Met compared with MHA-Ca. Therefore, the objective of the experiments presented was to determine the RBV of MHA-Ca compared with dl-Met to support N-retention in 15- to 21-kg starter pigs and to determine whether pigs demonstrated a preference for different sources of Met.

## Materials and Methods

Three experiments were conducted using nursery pigs. The first experiment was conducted at the Institute of Agricultural Sciences for Southern Vietnam under experimental protocols approved by the Animal Care Committee of the Institute. The second and third experiments were conducted at the University of Kentucky under experimental protocols approved by the Institutional Animal Care and Use Committee of the University of Kentucky. Prior to diet formulation, ingredients contributing AA were analyzed for AA composition and the analyzed AA contents and the standardized ileal digestibility coefficients according to AminoDat (3.0) were used in diet formulation in all experiments. Both test Met sources, dl-Met and MHA-Ca, were provided by Evonik Operations GmbH, and results of analyses confirmed the concentrations of 99.7 and 84% active substance in dl-Met and MHA-Ca, respectively.

### Experiment 1: animals and dietary treatments

A total of 42 crossbred [Duroc × (Large white/Landrace)] barrows with an initial body weight (**BW**) of 15.0 ± 0.7 kg were used in an N-balance trial with 2 groups of 21 pigs each. Each group of pigs provided 3 complete replicates (blocks) of the experimental diets. The basal diet (**BD**; Table 1) was formulated to be adequate in all essential nutrients except for Met (0.22% total Met), which was about 70% of the total Met requirement of 0.30% recommended by NRC (1998) for pigs weighing 10 to 20 kg. The Met-deficient BD was then supplemented with 3 graded levels of either dl-Met (0.025%, 0.050%, and 0.075%, as-fed basis) or MHA-Ca (0.038%, 0.077%, and 0.115%) at the expense of corn starch. The corresponding supplementation levels of MHA-Ca were based on an assumed average RBV of MHA-Ca to dl-Met of 65% (product-to-product basis) to provide similar animal performance. Supplemental AA other than Met were added to the BD to exceed the requirement for AA other than Met and Met + Cys (NRC, 1998).

**Table 1.** Experimental design and product analysis

	Experimental 1			Experimental 2		
Met source	Addition level, %	Analyzed content, %	Met bioequivalent, %	Addition level, %	Analyzed content, %	Met bioequivalent, %
Basal	—	—	—	—	—	—
dl-Met^1^	0.025	0.020	0.025	0.030	0.030	0.030
dl-Met	0.050	0.040	0.050	0.060	0.060	0.060
dl-Met	0.075	0.070	0.075	—	—	—
MHA-Ca^1^	0.038	0.030	0.025^2^	0.046	0.043	0.030^2^
MHA-Ca	0.077	0.060	0.050^2^	0.092	0.088	0.060^2^
MHA-Ca	0.115	0.100	0.075^2^	—	—	—

^1^ dl-Met = dl-methionine (99%); MHA-Ca = calcium salt of hydroxy analog of dl-Met (84%). Dietary additions were on an as-fed basis. The BD contained only intact protein and neither of the products evaluated. The analyzed contents of the diets are of the product that was added for the respective dietary treatments.

^2^Based on the relative bioavailability value of MHA-Ca to dl-Met of 65% (product-to-product basis) to provide an amount of MHA-Ca that would provide a presumed approximately equal animal performance.

### Experiment 2: animals and dietary treatments

A total of 40 barrows [Hampshire × (Landrace × Yorkshire)], with an initial BW of 15.5 ± 1.5 kg, obtained from the University of Kentucky Swine Research herd, were used in an N-balance trial with 2 groups of 20 pigs each. Each group of pigs included 4 complete replicates (blocks) of the experimental diets. The Met-deficient BD was formulated to contain 17.0% CP and 0.22% Met (Table 2) based on analyzed CP and AA of the ingredients. Five experimental diets included (1) BD, (2) BD + 0.030% dl-Met, (3) BD + 0.060% dl-Met, (4) BD + 0.046% MHA-Ca, and (5) BD + 0.092% MHA-Ca. The supplemental levels of MHA-Ca were based on an assumed average RBV of MHA-Ca to dl-Met of 65% (product-to-product basis) to provide similar animal performance. To prevent unintended variations from potential weighing or mixing errors, experimental diets were prepared by adding both Met products to a single common batch of the BD.

**Table 2.** Ingredient and nutrient composition of the BD (%, as-fed basis)

Item	Experiment 1	Experiment 2	Experiment 3
Ingredient, %			
Corn	72.57	68.00	56.00
Soybean meal, 48% CP	10.54	4.50	4.50
Dried whey	8.00	10.00	10.00
Spray-dried animal plasma	4.57	10.00	7.00
Pea	—	—	15.00
Corn oil	—	2.00	2.00
Cornstarch	1.00	2.46	2.26
Lysine·HCl	0.49	0.40	0.35
Thr	0.10	0.07	0.10
Trp	0.08	0.02	0.04
Ile	0.12	0.20	0.20
Val	0.06	—	—
Dicalcium phosphate	1.01	1.10	1.10
Ground limestone	0.96	0.95	0.95
Salt	0.25	—	0.20
Mineral–vitamin premix^1^	0.25	—	—
Vitamin premix^2^	—	0.10	0.10
Trace–mineral premix^3^	—	0.08	0.08
Antimicrobial^4^	—	0.10	0.10
Antioxidant^5^	—	0.02	0.02
Calculated nutrient composition, %^6^			
CP	15.44	17.02	17.28
Lys	1.17	1.37	1.36
Met	0.22	0.22	0.23
Met + Cys	0.54	0.63	0.62
Thr	0.76	0.95	0.92
Trp	0.23	0.25	0.26
Ile	0.63	0.85	0.82
Val	0.79	1.02	0.93
Calculated energy content^7^			
ME, kcal/kg	3,260	3,450	3,400

^1^Supplied per kilogram of diet: 10,000 IU of vitamin A, 2000 IU of vitamin D_3_, 15 mg of vitamin E, 2 mg of vitamin K_3_, 1.3 mg of thiamin, 3.5 mg of riboflavin, 1.5 mg of pyridoxine, 0.025 mg of vitamin B_12_, 0.6 mg of folic acid, 15 mg of niacin, 10 mg of calcium pantothenate, 0.1 mg of d-biotin, 150 mg of Zn, 80 mg of Fe, 60 mg of Mn, 6 mg of Cu, 0.75 mg of I, 0.75 mg of Co, 0.10 mg of Se, and 150 mg of ethoxyquin.

^2^Supplied per kilogram of diet: 6600 IU of vitamin A, 880 IU of vitamin D_3_, 44 IU of vitamin E, 6.4 mg of vitamin K (as menadione sodium bisulfite complex), 4.0 mg of thiamin, 8.8 mg of riboflavin, 4.4 mg of pyridoxine, 33 µg of vitamin B_12_, 1.3 mg of folic acid, 44 mg of niacin, 22 mg of pantothenic acid, and 0.22 mg of d-biotin.

^3^Supplied per kilogram of diet: 140 mg of Zn as ZnO, 140 mg of Fe as FeSO_4_·H_2_O, 48 mg of Mn as MnO, 14 mg of Cu as CuSO_4_·5H_2_O, 1.6 mg of I as CaI_2_O_6_, 0.24 mg of Co as CoCO_3_, and 0.30 mg of Se as NaSeO_3_.

^4^Supplied 22 mg of carbadox per kilogram of diet as 0.1% of Mecadox-10 (2.2% carbadox, Phibro Animal Health, Fairfield, NJ).

^5^Supplied 130 mg of ethoxyquin per kilogram of diet as 0.02% of Santoquin (Novus International, St. Louis, MO).

^6^Calculated values for CP and AA of the BDs used in experiments 1 and 2 are based on the analyzed values of all protein-containing ingredients.

^7^Calculated values for ME are based on NRC (1998).

### General experimental design and procedure (experiments 1 and 2)

In both experiments 1 and 2, pigs were placed in metabolism crates for a 7-d adaptation to their diet and crate. Feed allowance was equalized within block at ~3.5% of the average BW. One-third of the daily feed allowance (in mash form) was provided at 0800, 1200, and 1600 hours in experiment 1 and at 0600, 1300, and 2000 hours in experiment 2 for the 7-d adaptation period and the 5-d collection period, mixed with a sufficient quantity of water to create a gruel. Feed allowance for the collection period was adjusted based on the final BW for the adaptation period. In experiment 1, after a 7-d adaptation period to the diets and metabolism crate, feces and urine were collected quantitatively for 5 d in a manner similar to Feng et al. (2006) starting after the morning feed allotment on day 8 and terminating shortly before the morning feed allotment on day 13. In experiment 2, the beginning and end of the collection period were marked by the addition of 0.5% indigo carmine (Adeola, 2001) to the morning feed allotment. After consumption of each meal, water was added to the metabolism crate feeder to allow ad libitum access to water between meals.

During the collection periods, the total quantities of feces excreted were collected daily, stored in plastic bags, and frozen at −20 °C until the end of the collection period. The total feces collected for 5 d were dried in a forced-air drying oven for 72 hr at 55 °C. The dried fecal samples were ground to pass a 1-mm screen in a Wiley Mill (Model 3; Arthur H. Thomas, Philadelphia, PA) for analysis of DM and nitrogen (N) content. The collection of urine was initiated 14 hr after feeding of the first marked meal and was completed 14 hr after feeding of the second marked meal at the end of the collection period. A total of 10 mL of 6 N HCl (experiment 1) and 150 mL of 3 N HCl (experiment 2), respectively, were added to the collection container at the beginning of each collection to prevent volatilization of urinary N. Urine was collected every 24 hr and stored at −20 °C. At the end of the collection phase, the total quantity of urine collected from each pig was allowed to thaw, then measured and pooled. Two aliquots (~200 mL) of urine from each pig were subsampled for N analysis. The average of the analyzed dietary values in experiment 2 was used for diet N content calculation because of the mixing of a common BD for all dietary treatments.

### Experiment 3: animals, dietary treatments and experimental procedure

A total of 276 barrows and gilts [Hampshire × (Landrace × Yorkshire)], with an initial BW of 7.09 ± 1.1 kg, were weaned at an average of 21 d of age (range of 18 to 25 d) and used in 3 4-wk diet preference studies to determine whether pigs preferred diets based on the diet Met content and the different Met sources. The BD (Table 2) was formulated to be adequate in all essential nutrients except for Met (0.23% Met), which was clearly below the Met requirements recommended by NRC (1998) for pigs weighing 5 to 10 kg and 10 to 20 kg (0.35% and 0.30% Met, respectively). Within each study, pigs were blocked by BW within gender and randomly allotted to 1 of 3 dietary treatment comparisons: (1) choice of BD or BD plus 0.07% dl-Met; (2) choice of BD or BD plus 0.0825% MHA-Ca, and (3) choice of BD plus either 0.07% dl-Met or 0.0825% MHA-Ca. The pigs were housed 4 pigs per pen for a total of 23 replicates (60 pigs, 5 replicates from study 1; 120 pigs, 10 replicates from study 2; 96 pigs, 8 replicates from study 3; these 23 replicates were comprised of 12 barrow and 11 gilt replicates).

Two feeders, each with one of the two diets in the treatment comparison, were placed in each pen and the location of the feeders was rotated each Monday, Wednesday, and Friday to avoid the potential of feeder location being confounded with potential feed preference exhibited. Pigs were given ad libitum access to feed and water (diet composition is provided in Table 2).

### Laboratory analyses

The DM in feed and feces was determined after oven-drying for 4 hr at 103 °C (AOAC, 2000). The N content of the diets, feces, and urine was determined using a gas combustion method (AOAC, 1998; FP-2000, Leco Corp., St. Joseph, MI). Ethylenediaminetetraacetic acid was used as a reference standard before and after all N analyses. Dietary AA concentrations were determined by ion-exchange chromatography with postcolumn derivatization with ninhydrin. AA were oxidized with performic acid, which was neutralized with sodium metabisulfite (Llames and Fontaine, 1994; European Community, 1998). Briefly, AA were liberated from the protein by hydrolysis with 6 N HCl for 24 hr at 110 °C and quantified with the internal standard method by measuring the absorption of reaction products with ninhydrin at 570 nm. Tryptophan was determined by HPLC with fluorescence detection (extinction 280 nm, emission 356 nm) after alkaline hydrolysis with barium hydroxide octahydrate for 20 hr at 110 °C (European Community, 2000). Tyrosine was not determined. Supplemented AA were determined after extraction with 0.1 N HCl (European Community, 1998). Supplemented MHA-Ca was analyzed using the method described by VDLUFA (1997).

### Statistical analyses

#### Experiments 1 and 2

 The experimental data were analyzed as a randomized complete block design using GLM procedures of SAS (SAS Inst., Inc., Cary, NC) with individual pig considered the experimental unit. The statistical model included treatment, group, and block (group). Orthogonal polynomial contrasts were conducted to determine linear and quadratic effects of increasing the graded levels of dl-Met and MHA-Ca on response criteria. Due to the linear response in both studies and having only 3 points in response curve in experiment 2, the RBV of the 2 Met sources was determined by the multivariate linear regression model based on N retained (treatment means) as a response of supplemental Met level (%) using the following equation:

$$\begin{array}{l} y=a+\;b_{1}x_{1}+\;b_{2}x_{2} \end{array}$$

in which *y* = response criterion (N retained); *a* = intercept; *b*_1_, *b*_2_ = the slope of dl-Met and MHA-Ca, respectively; *x*_1_, *x*_2_ = dietary inclusion level (%) of dl-Met and MHA-Ca, respectively. The RBV of MHA-Ca when compared with dl-Met was calculated as the ratio of their linear slopes (i.e., *b*_2_/*b*_1_ × 100) as described by Littell et al. (1997). Differences were considered significant if *P* < 0.05 and tendencies if 0.05 ≤ *P* < 0.10.

#### Experiment 3

Feed disappearance values were converted to percentage consumption of each diet. The percentages for each period and for the cumulative time periods were subjected to unpaired *t*-test using Graph Pad Prism (Graph Pad Software, San Diego, CA) with the pen considered the experimental unit. Differences were considered significant if *P* < 0.05 and tendencies if 0.05 ≤ *P* < 0.10.

## Results

Analysis of the experimental diets confirmed that the contents of supplemental dl-Met and MHA-Ca were very close to the formulated levels. The analyzed contents of AA including the supplemented dl-Met and MHA-Ca were slightly lower than the calculated values across the experimental diets. However, the corresponding dl-Met to MHA-Ca ratio of ~65% (wt/wt) was maintained in the Met-supplemented diets (Tables 1 and 2).

### Experiment 1

The BD contained 0.22% Met, and the added levels of dl-Met and MHA-Ca were analyzed to be 0.020%, 0.040%, and 0.070% dl-Met; 0.030%, 0.060%, and 0.100% MHA-Ca, which are close to calculated values (Table 1). The effects of graded levels of dl-Met or MHA-Ca on N retention by pigs are presented in Table 3. An increase in the level of supplemental dl-Met and MHA-Ca in the Met-deficient BD from 0% to 0.075% and 0% to 0.115%, respectively, linearly decreased (*P* < 0.001) fecal N output per day (g). Urinary N excretion per day (g) was linearly decreased (*P* = 0.001) with graded levels of dietary dl-Met from 0% to 0.075%. Increasing dietary inclusion rate of both Met sources resulted in a linear increase (*P* < 0.01) in retained N per day (g) and N retention rate (% of intake). Quadratic effects of including dietary Met sources on retained N or N retention rate were not observed.

**Table 3.** Effects of supplementation with graded level of DL-Met or MHA-Ca on BW and N retention of pigs in experiment 1^1,2^

Added source			dl-Met			MHA-Ca				Linear *P*-value^8^		Quadratic *P*-value^9^	
Item	Level, %	BD	0.025	0.050	0.075	0.038	0.077	0.115	SEM	dl-Met	MHA-Ca	dl-Met	MHA-Ca
Pig weight, kg													
Beginning of adaptation		15.08	14.82	15.03	15.05	14.92	15.08	15.17	0.671	0.969	0.890	0.834	0.853
End of adaptation		15.95	15.75	16.15	16.08	16.00	16.25	16.33	0.675	0.793	0.646	0.922	0.981
End of collection		17.33	17.15	17.53	17.50	17.42	17.75	17.83	0.666	0.769	0.542	0.911	1.000
N intake, g/d^3^		14.95	14.48	14.86	14.31	14.59	14.49	15.13	0.215	0.114	0.653	0.841	0.026
Fecal N, g/d^4^		1.95	1.92	1.77	1.61	1.74	1.63	1.54	0.056	<0.001	<0.001	0.228	0.276
Urine N, g/d^5^		2.66	1.95	2.09	1.33	2.17	1.97	2.22	0.202	0.001	0.104	0.908	0.676
N retained, g/d^6^		10.34	10.61	11.00	11.37	10.69	10.89	11.37	0.227	0.002	0.003	0.824	0.765
N retention, % of intake^7^		69.35	73.42	74.59	79.55	73.36	75.62	75.17	1.295	<0.001	0.002	0.733	0.094

^1^DL-Met = dl-Met (99%); MHA-Ca = calcium salt of hydroxy analog of dl-Met (84%).

^2^Each mean represents 6 individually housed barrows.

^3^N intake, g/d = daily feed consumption during the collection period, g/d × (feed N content, % ÷ 100).

^4^Fecal N, g/d = feces weight, g, air-dry basis × (feces N content, % ÷ 100)/5 d.

^5^Urine N, g/d = urine weight, g × (urine N content, % ÷ 100)/5 d.

^6^N retained, g/d = (N intake, g − fecal N, g − urine N, g)/5 d.

^7^N retention, % = (N retained, g ÷ N intake, g) × 100.

^8^Linear = linear effects of added dl-Met and MHA-Ca diets utilized the BD (0% added product) and the 3 added levels of the respective products to determine the orthogonal contrast coefficients.

^9^Quadratic = quadratic effects of added dl-Met and MHA-Ca diets utilized the BD (0% added product) and the 3 added levels of the respective products to determine the orthogonal contrast coefficients.

**Figure 1.**
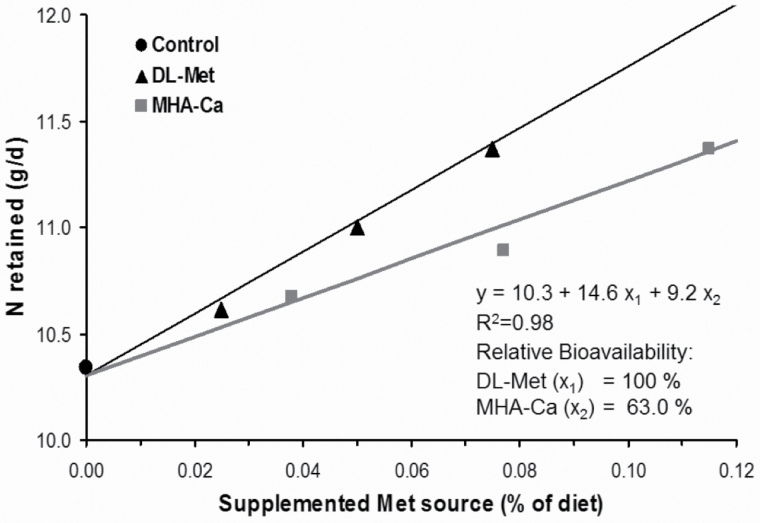
Bioavailability of the calcium salt of hydroxy analog of dl-Met (MHA-Ca) relative to dl-Met based on N retained (g/d) in experiment 1. Relative bioavailability of MHA-Ca to dl-Met for N retention was 63.0% (9.2/14.6 × 100) on a product basis.

Using a linear slope-ratio procedure for comparison of the 2 Met sources, a product-to-product RBV of MHA-Ca compared with dl-Met of 63.0% was observed based on N retained expressed as gram per day (Figure 1). Based on an MHA-Ca content of 84% in the commercial product, this equates to an RBV of MHA-Ca to dl-Met of 75.0% on an equimolar basis.

### Experiment 2

The BD contained 0.22% Met, and the added levels of dl-Met and MHA-Ca were analyzed to be 0.030% and 0.060% dl-Met; 0.043% and 0.088% MHA-Ca, confirming that the inclusion levels of both Met sources were as expected (Table 1). The effects of including graded levels of dl-Met and MHA-Ca in Met-deficient diet for pigs on N retention are presented in Table 4. Dietary supplementation of both Met sources to the Met-deficient BD diet did not affect fecal N output per day (g). However, an increase in dietary level of dl-Met and MHA-Ca from 0% to 0.060% and 0% to 0.092%, respectively, resulted in a linear decrease (*P* < 0.001) in urinary N excretion per day (g), whereas retained N per day (g) linearly increased (*P* < 0.001) with graded levels of both Met sources. Increasing graded levels of dl-Met and MHA-Ca from 0% to 0.060% and 0% to 0.092%, respectively, linearly increased (*P* < 0.001) N retention rate (% of intake). In addition, quadratic effects of supplemental Met sources on retained N or N retention rate (*P* < 0.05) were observed.

**Table 4.** Effects of supplementation with graded level of dl-Met or MHA-Ca on BW and N retention of pigs in experiment 2^1,2^

Added source			DL-Met		MHA-Ca			Linear *P*-value^8^		Quadratic *P*-value^9^	
Item	Level, %	BD	0.030	0.060	0.046	0.092	SEM	DL-Met	MHA-Ca	DL-Met	MHA-Ca
Pig weight, kg											
Beginning of adaptation		15.49	15.88	15.62	15.05	15.38	0.248	0.719	0.753	0.293	0.212
End of adaptation		17.35	18.11	17.82	17.20	17.54	0.307	0.284	0.665	0.174	0.529
End of collection		20.37	21.34	20.84	20.48	20.79	0.331	0.327	0.381	0.080	0.800
N intake, g/d^3^		16.81	16.84	16.84	16.83	16.84	0.016	0.149	0.149	0.398	0.778
Fecal N, g/d^4^		1.46	1.20	1.30	1.36	1.41	0.073	0.125	0.599	0.050	0.398
Urine N, g/d^5^		5.12	4.09	3.66	3.85	3.49	0.164	<0.001	<0.001	0.151	0.032
N retained, g/d^6^		10.23	11.55	11.88	11.62	11.94	0.183	<0.001	<0.001	0.035	0.024
N retention, % of intake^7^		61.40	69.13	71.15	69.53	71.53	1.083	<0.001	<0.001	0.039	0.028

^1^ dl-Met = dl-Met (99%); MHA-Ca = calcium salt of hydroxy analog of dl-Met (84%).

^2^Each mean represents 8 individually housed barrows.

^3^N intake, g/d = daily feed consumption during the collection period, g/d × (feed N content, % ÷ 100).

^4^Fecal N, g/d = feces weight, g, air-dry basis × (feces N content, % ÷ 100)/5 d.

^5^Urine N, g/d = urine weight, g × (urine N content, % ÷ 100)/5 d.

^6^N retained, g/d = (N intake, g − fecal N, g − urine N, g)/5 d.

^7^N retention, % = (N retained, g ÷ N intake, g) × 100.

^8^Linear = linear effects of added dl-Met and MHA-Ca diets utilized the BD (0% added product) and the 2 added levels of the respective products to determine the orthogonal contrast coefficients.

^9^Quadratic = quadratic effects of added dl-Met and MHA-Ca diets utilized the BD (0% added product) and the 2 added levels of the respective products to determine the orthogonal contrast coefficients.

**Figure 2.**
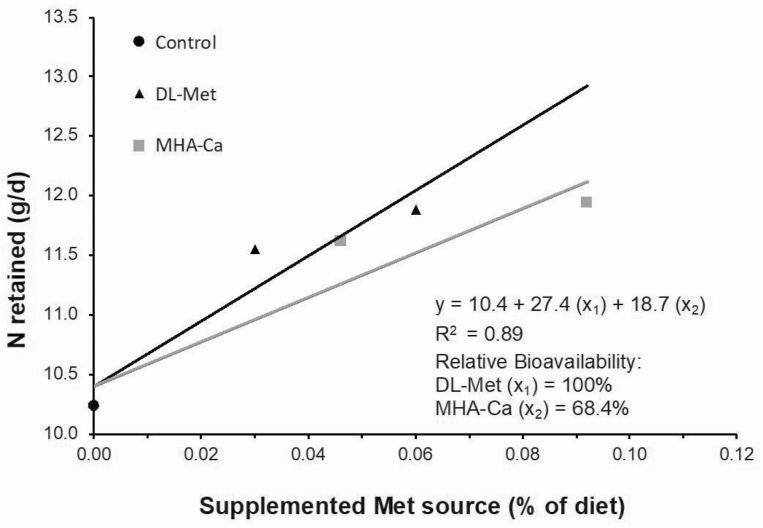
Bioavailability of the calcium salt of hydroxy analog of dl-Met (MHA-Ca) relative to dl-Met based on N retained (g/d) in experiment 2. Relative bioavailability of MHA-Ca to dl-Met for N retention was 68.4% (18.7/27.4 × 100) on a product basis.

The linear slope-ratio regression estimated a product-to-product relative biological equivalence value of MHA-Ca to dl-Met in the current experiment was 68.4% based on N retained expressed in gram per day (Figure 2), which equates to an RBV value of MHA-Ca to dl-Met of 81.4% on an equimolar basis.

### Experiment 3

The diet preferences of pigs for the comparison of the Met-deficient BD relative to dl-Met-supplemented diet are presented in Table 5. The results of the current study revealed that no diet preference was exhibited by pigs during the first 14 d. However, pigs preferred to consume more of the dl-Met-supplemented diet (*P* < 0.05) than the BD for days 14 to 21 and 21 to 28 periods. Based on the percentage of feed consumption during the periods (from days 14 to 21 and 21 to 28), there was a pronounced preference (*P* < 0.01) in cumulative consumption for the diet containing 0.07% dl-Met. At the termination of the study on day 28, the pigs chose to consume more of the dl-Met-supplemented diet, with the ratio of 0.07% dl-Met to Met-deficient BD being ~55:45.

**Table 5.** Average daily feed intake (g) and individual period and cumulative feed preference (%) exhibited by nursery pigs when offered a choice of 2 diets with either 0% or 0.07% dl-Met in experiment 3^1^

	dl-Met			0% vs. 0.07% DL-Met	
Days	0% (basal)	0.07%	SEM	For the period, % consumed	Cumulative consumption
0 to 7	178	218	31	47.8 vs. 52.2	
7 to 14	305	333	27	48.6 vs. 51.4	48.1 vs. 51.9
14 to 21	405	542	39	43.3 vs. 56.7^**^	45.9 vs. 54.1^**^
21 to 28	499	633	57	44.5 vs. 55.5*	45.4 vs. 54.6^**^

^1^Each mean represents 23 observations per treatment.

*P < 0.05; ^**^P < 0.01.

The diet preferences of pigs for the comparison of the Met-deficient BD compared with 0.0825% MHA-Ca are provided in Table 6. On days 7, 21, and 28, pigs preferred to consume less of the MHA-Ca-supplemented diet than that of the BD (*P* < 0.05). By the conclusion of the study on day 28, pigs consumed less of the MHA-Ca-fortified diet (*P* < 0.05) with a ratio of ~55:45. The diet preferences of pigs for the comparison of 0.07% dl-Met relative to 0.0825% MHA-Ca are shown in Table 7. The results demonstrated that no diet preference for 0.07% dl-Met and 0.0825% MHA-Ca existed during any of the study periods.

**Table 6.** Average daily feed intake (g) and individual period and cumulative feed preference (%) exhibited by nursery pigs when offered a choice of 2 diets with either 0 or 0.0825% MHA-Ca in experiment 3^1^

	MHA-Ca			0% vs. 0.0825% MHA-Ca	
Days	0% (basal)	0.0825%	SEM	For the period, % consumed	Cumulative consumption
0 to 7	239	164	31	57.5 vs. 42.5^**^	
7 to 14	343	307	30	52.7 vs. 47.3	54.4 vs. 45.6^**^
14 to 21	530	407	39	56.6 vs. 43.4^**^	55.3 vs. 44.7^**^
21 to 28	622	509	45	55.0 vs. 45.0*	55.2 vs. 44.8^**^

^1^Each mean represents 23 observations per treatment.

*P < 0.05; ^**^P < 0.01.

**Table 7.** Average daily feed intake (g) and individual period and cumulative feed preference (%) exhibited by nursery pigs when offered a choice of 2 diets with either 0.07% dl-Met or 0.0825% MHA-Ca in experiment 3^1^

	dl-Met	MHA-Ca		0.07% dl-Met vs. 0.0825% MHA-Ca	
Days	0.07%	0.0825%	SEM	For the period, % consumed	Cumulative consumption
0 to 7	209	209	24	50.7 vs. 49.3	
7 to 14	372	370	20	50.2 vs. 49.8	50.4 vs. 49.6
14 to 21	521	488	31	51.7 vs. 48.3	50.8 vs. 49.2
21 to 28	584	563	44	51.1 vs. 48.9	50.9 vs. 49.1

^1^Each mean represents 23 observations per treatment.

*P < 0.05; ^**^P < 0.01.

The feed intake preferences of pigs for diets supplemented with either 0.07% dl-Met or 0.0825% MHA-Ca were further divided into evaluations of the different gender response and are provided in Table 8. During the overall period (days 0 to 28), in comparison 1 barrows preferred to consume more of the dl-Met-supplemented diet (*P* < 0.05) than the Met-deficient BD, whereas no diet preference was exhibited by gilts. Barrows in 8 of 12 pens consumed more of the dl-Met-supplemented diet, with the ratio of dl-Met to Met-deficient BD being ~57:43. For the total 28-d study for comparison 2, barrows consumed less of the MHA-Ca-supplemented diet than the BD (*P* < 0.05), whereas gilts exhibited no diet preference. Barrows in only 2 of 12 pens consumed more of the MHA-Ca-fortified diet, with the ratio of Met-deficient BD to MHA-Ca being ~60:40. No preference differences were observed for either the barrow or gilt replicates for the comparison of 0.07% dl-Met relative to 0.0825% MHA-Ca in comparison 3.

**Table 8.** Average daily feed intake (g) and cumulative feed preference (%) exhibited by nursery pigs when offered a choice of 2 diets with either 0 or 0.07% dl-Met; 0 or 0.0825% MHA-Ca; 0.07% dl-Met or 0.0825% MHA-Ca in experiment 3^1^

	dl-Met		MHA-Ca		
Item	0%	0.07%	0.0825%	SEM	Cumulative consumption^2,3,4^
Comparison 1 (0 vs. 0.07% dl-Met)					
Days 0 to 28 (all replicates)	347	431	—	33	45.4 vs. 54.6*(14/23)
Barrows	341	479	—	55	43.1 vs. 56.9*(8/12)
Gilts	353	379	—	31	48.0 vs. 52.0 (6/11)
Comparison 2 (0 vs. 0.0825% MHA-Ca)					
Days 0 to 28 (all replicates)	433	—	347	31	55.2 vs. 44.8^**^(8/23)
Barrows	475	—	303	52	60.2 vs. 39.8^**^(2/12)
Gilts	388	—	395	20	49.8 vs. 50.2 (6/11)
Comparison 3 (0.07% dl-Met vs. 0.0825% MHA-Ca)					
Days 0 to 28 (all replicates)	—	422	408	22	50.9 vs. 49.1 (10/23)
Barrows	—	429	400	35	51.9 vs. 48.1 (5/12)
Gilts	—	414	416	28	49.9 vs. 50.1 (5/11)

^1^Each mean represents 23 observations per treatment for all replicates, which is comprised of 12 replicates of barrows and 11 replicates of gilts.

^2^Values presented in parentheses are the number of pens from a total of 23 pens (or 12 pens of barrows or 11 pens of gilts) that consumed more of the diet with 0.07% dl-Met.

^3^Values presented in parentheses are the number of pens from a total of 23 pens (or 12 pens of barrows or 11 pens of gilts) that consumed more of the diet with 0.0825% MHA-Ca.

^4^Values presented in parentheses are the number of pens from a total of 23 pens (or 12 pens of barrows or 11 pens of gilts) that consumed more of the diet with 0.0825% MHA-Ca.

*P < 0.05; ^**^P < 0.01.

## Discussion

The primary response criterion of the current study was N retention in pigs. The BD was formulated to be deficient in Met, which is a prerequisite, to determine the RBV of MHA-Ca to dl-Met using the slope-ratio procedure (Littell et al., 1997). Based on the results for the analyzed content of Met in the BD coupled with a clearly lower N retention in pigs fed BD, this criterion was clearly met.

The supplementation of the Met-deficient BD with graded levels of either dl-Met or MHA-Ca resulted in a linear improvement in N retention by pigs. The increase in N retention due to supplemental Met sources is attributed to the utilization of other AA as the Met deficiency was alleviated as evidenced by the linear reduction in urinary N excretion for dl-Met and MHA-Ca. The improvement in dietary N utilization by pigs due to supplemental Met sources is in agreement with the results from the study of Opapeju et al. (2012) who reported linearly reduced urinary N excretion and improved N retention in growing pigs fed Met-deficient diet supplemented with incremental levels of MHA-Ca. In the study of Zimmermann et al. (2005), supplemental MHA-FA at 0.0855% resulted in a reduction in urinary N excretion, leading to increased N retention by growing pigs fed a Met-deficient diet. Furthermore, Kim et al. (2006) reported a linear reduction in urinary N excretion by growing pigs due to supplementation of a Met-deficient diet with increasing levels of MHA-FA as a result of increasing dietary N retention demonstrating the well-established fact that supplemental Met sources improve N utilization of the Met-deficient diet by pigs.

The mean value of the RBV of MHA-Ca to dl-Met on a product-to-product basis was 65.7% (63.0 and 68.4% for experiments 1 and 2, respectively) based on the N retention per day (g), resulting in the lower biological efficacy of MHA-Ca than that of dl-Met of 78.2% on an equimolar basis. The lower RBV of MHA-Ca may have been due, as postulated by Opapeju et al. (2012) to the lower rate of the biochemical conversion of MHA-Ca to l-Met compared with that of dl-Met to l-Met by pigs. To be utilized for protein synthesis, both the d- and l-isomers of MHA-Ca must be converted by l-hydroxy acid oxidase, d-hydroxy acid dehydrase, and d-amino acid oxidase, respectively, into keto-Met via oxidation, followed by transamination into the bioactive l-Met (Dibner, 2003; Dilger and Baker, 2008). dl-Met is composed of 50% d-Met and 50% l-Met and only the d-isomer of dl-Met must be converted. In addition, Chung and Baker (1992) reported 100% molar efficiency of d-Met conversion to l-Met by weanling pigs, implying that the efficiency of the utilization of dl-Met by pigs can be greater than that of MHA-Ca, which is more slowly absorbed throughout the digestive tract associated with more exposure to microbial degradation in the small intestine and less available for absorption (Malik et al., 2009). The lower bioefficacy of MHA-Ca relative to dl-Met is in agreement with the results from the study of Opapeju et al. (2012) who reported a relative bioequivalence value of MHA-Ca to dl-Met of 71.2% on a product-to-product basis in growing pigs weighing from 18 to 22 kg for N retention expressed as % of intake, which equates to 84.8% on an equimolar basis. Shoveller et al. (2010) assessed the biological efficacy for MHA-FA compared with dl-Met in growing pigs using the indicator AA oxidation method in a slope-ratio comparison. The RBV of MHA-FA to dl-Met for protein deposition was 65.7% on a product-to-product basis, which equates to 74.4% on an equimolar basis. In the review article of Jansman et al. (2003) that reported the biological efficacy for MHA-FA relative to dl-Met, the mean RBV of MHA-FA to dl-Met in growing pigs was 72.2% on a product-to-product basis, which equates to 82% on an equimolar basis. Also, Zimmerman et al. (2005) observed a relative bioequivalence value of MHA-FA to dl-Met of 62.0% on a product-to-product basis in growing pigs for N retention (g/d), which equates to 70.5% on an equimolar basis. In the study of Kim et al. (2006), the RBV of MHA-FA to dl-Met in growing pigs weighing from 16 to 21 kg was 65.3% on a product-to-product basis, which equates to 74.1% on an equimolar basis for N retention. Furthermore, Feng et al. (2006) reported the RBV for MHA-FA to dl-Met was 73.2% on a product-to-product basis in growing pigs, which equates to 83.2% on an equimolar basis for N retention.

In the current study, pigs preferred to consume more of the dl-Met-supplemented diet at 0.07% than the BD. The diet preference of pigs for the Met-fortified diets over the Met-deficient diets could be attributed to the distinctive ability of pigs to identify AA deficiency in diets. However, the mechanisms by which pigs exhibit a preference for Met-adequate diets over Met-deficient diets and the main reason for this response by pigs are unknown. The results of the current study are in agreement with the results reported in the study of Roth et al. (2006), who observed a clear preference of nursery pigs for Met-fortified diets over Met-deficient diets. Also, the concept of the preference being attributable to an amino acid deficiency is supported by the work of Ettle and Roth (2004) who reported that nursery pigs preferred to consume more of the Trp-sufficient diets than Trp-deficient diets. In another study conducted by Ettle and Roth (2009), results demonstrated a preference of nursery pigs for Lys-sufficient diets over Lys-deficient diets, again implying that nursery pigs are capable of detecting deficiency in AA when given a choice of diets adequate or deficient in AA. However, this concept is not supported by the present data that showed a discrimination against a diet whose Met adequacy was accomplished by supplementation with MHA-Ca. Paradoxically, in the third preference comparison when dl-Met and MHA-Ca diets were compared directly, where the results of the first two comparisons would suggest extreme discrimination against MHA-CA supplemented diets, there was no preference exhibited. The differences in preference for Met sources were exhibited strictly in barrows but not in gilts. To our knowledge, this is the first report that demonstrates a gender-specific preference for Met sources. Thus, further research is warranted to investigate preference of nursery pigs for various Met sources by gender.

In conclusion, supplementation of Met-deficient diets with MHA-Ca or dl-Met resulted in significant improvements in N retention by pigs as evidenced by reductions in urinary N excretion. The results of the current studies demonstrated that the mean RBV of MHA-Ca to dl-Met for N retention was 65.7% on a product-to-product basis, which equates to 78.2% on an equimolar basis. When given a choice, pigs preferred the diet supplemented with dl-Met more than the diet with no supplemental Met. In contrast, pigs showed a preference for the diet without supplemental Met over the diet supplemented with MHA-Ca. Barrows exhibited a stronger preference than gilts for the source of supplemental Met in the diets.

## Abbreviations

BD: basal diet
BW: body weight
dl-Met: dl-methionine
MHA-Ca: calcium salt of hydroxy analog of dl-methionine
MHA-FA: dl-methionine hydroxy analog-free acid
RBV: relative bioavailability
